# Isolation, Purification, and Characterization of Xylanase Produced by a New Species of *Bacillus* in Solid State Fermentation

**DOI:** 10.1155/2012/683193

**Published:** 2012-01-17

**Authors:** Rajashri D. Kamble, Anandrao R. Jadhav

**Affiliations:** ^1^Department of Biotechnology Engineering, Tatyasaheb Kore Institute of Engineering & Technology, Warananagar Panhala, Kolhapur, Maharashtra, 416113, India; ^2^Department of Microbiology, K.R.P. Kanya Mahavidyalaya, Islampur, Walwa, Sangli, Maharashtra, 415414, India

## Abstract

A thermoalkalophilic new species of *Bacillus*, similar to *Bacillus arseniciselenatis* DSM 15340, produced extracellular xylanase under solid state fermentation when wheat bran is used as carbon source. The extracellular xylanase was isolated by ammonium sulfate (80%) precipitation and purified using ion exchange chromatography. The molecular weight of xylanase was ~29.8 kDa. The optimum temperature and pH for the enzyme activity were 50°C and pH 8.0. The enzyme was active on birchwood xylan and little active on *p*-nitrophenyl xylopyranoside but not on Avicel, CMC, cellobiose, and starch, showing its absolute substrate specificity. For birchwood xylan, the enzyme gave a Km 5.26 mg/mL and Vmax 277.7 **μ**mol/min/mg, respectively. In addition, the xylanase was also capable of producing high-quality xylo-oligosaccharides, which indicated its application potential not only in pulp biobleaching processes but also in the nutraceutical industry.

## 1. Introduction

Xylan is the most abundant noncellulosic polysaccharide present in both hardwoods and annual plants and accounts for 20–35% of the total dry weight in tropical plant biomass [1–3]. In temperate softwoods, xylans are less abundant and may comprise about 8% of the total dry weight [4]. Xylan is found mainly in the secondary cell wall and is considered to be forming an interphase between lignin and other polysaccharides. It is likely that xylan molecules covalently link with lignin phenolic residues and also interact with polysaccharides, such as pectin and glucan. In simplest forms, xylans are linear homopolymers that contain D-xylose monomers linked through *β*-1, 4–glycosyl bonds [5, 6]. Xylanase (E.C 3.2.1.8) degrades *β*-1, 4 xylan by cleaving *β*-1, 4 glycosidic linkages randomly, and the products are xylose and xylo-oligosaccharides like xylobiose [7, 8]. Xylanases are of industrial importance, which can be used in paper manufacturing to bleach paper pulp, increasing the brightness of pulp and improving the digestibility of animal feed and for clarification of fruit juices. Applications of xylanase avoid the use of chemicals that are expensive and cause pollution [9]. Microorganisms are the rich sources of xylanases, produced by diverse genera and species of bacteria, actinomycetes, and fungi. Several species of *Bacillus* and filamentous fungi secrete high amounts of extracellular xylanases [10]. Xylanase secretion often associates with low or high amount of cellulases. To use xylanase for pulp treatment, it is preferable to use cellulose-free xylanases, since the cellulase may adversely affect the quality of the paper pulp [11–15]. The most practical approach is the screening for naturally occurring microbial strains capable of secreting cellulose-free xylanases under optimized fermentation conditions. To use xylanase prominently in bleaching process it should be stable at high temperature and alkaline pH [16, 17]. 

Industrial production of enzymes on large scale is associated mainly with substrate. The use of agriculture residues as low-cost substrates for the production of industrial enzymes is a significant way to reduce production cost. The technique of fermentation using solid state substrate has the great advantage over submerged fermentation due to absence or near absence of aqueous phase that provides natural habitat for growth of microorganisms, economy of the space, simplicity of the media, no complex machinery, equipments and control systems, greater compactness of the fermentation vessel owing to a lower water volume, greater product yields, reduced energy demand, lower capital and recurring expenditures in industry, easier scale-up of processes, lesser volume of solvent needed for product recovery, superior yields, absence of foam build-up, and easier control of contamination due to the low moisture level in the system [10, 18]. In consideration with these facts the present study aims to characterize extracellular alkalothermophilic xylanase produced by *Bacillus arseniciselenatis *DSM 15340 when grown in solid state fermentation. To our knowledge, this is the first report describing the production of thermoalkalophilic cellulase-free xylanase by *Bacillus arseniciselenatis *DSM 15340. In addition, this xylanase was found to be able to degrade xylan into xylo-oligosaccharides.

## 2. Materials and Methods

### 2.1. Screening of Xylanolytic Strains

Soil samples were collected from coastal areas of Mandovi, Goa, India. Enrichment was done using birchwood xylan (Sigma Chemicals, Germany) as a sole source of carbon. Twenty five bacterial cultures were screened for xylanolytic ability by adding dye-labelled substrate, for example, xylan-brilliant red 3BA in xylan agar medium [19].

### 2.2. Phenotypic Characteristics

Prominent selected isolate was identified on the basis of morphological, cultural, biochemical properties [20] and 16S rRNA sequencing. Culture was deposited at National Centre for Cell Sciences (NCCS), Pune, India.

### 2.3. Phylogenic Analysis

The partial 16S rRNA sequences were retrieved on NCBI server (http://blast.ncbi.nlm.nih.gov/Blast.cgi) using BLAST tool. Top 10 similar sequences were downloaded in FASTA format. Multiple alignment of sequences and calculations of levels of sequence similarity were performed by using ClustalW2 program. A phylogenetic tree obtained was analyzed for closely related organism. The evolutionary history was inferred using the neighbor-joining method [14].

### 2.4. Growth Conditions of Culture

The bacterial isolate was maintained in liquid medium as well as solid medium in basal salt solution (BSS) containing 0.5% xylan having pH 8.0 at 45°C and stored at 4°C.

### 2.5. Xylanase Production in Solid State Fermentation (SSF)

The selected strain was further tested for their abilities to produce extracellular xylanase under solid state fermentation. Wheat bran was used as the substrate. For this the strain was cultured in Erlenmeyer flasks (250 mL) containing 10 g of wheat bran moistened with 18 mL of the basal salt solution (BSS: substrate-to-moisture ratio 1 : 1. 8). After 48 h of fermentation spent; solid substrate was removed and suspended in 50 mM phosphate buffer (pH 8.0), vortexed thoroughly to extract the xylanase. The sample was centrifuged at 5000 ×g for 10 minutes at 4°C. Centrifugation will remove xylanase from substrate. Supernatant was filtered through Whatman No. 1 filter paper and the clear filtrate was used as crude xylanase preparation. Prior to centrifugation, the samples were withdrawn for determining viable number of cells by standard viable plate count technique.

### 2.6. Xylanase Assay

Xylanase activity was measured according to Bailey et al. [21]. A 900 *μ*L of 1% solubilised birchwood xylan solution was added with 100 *μ*L enzyme solution in a test tube. 1.5 mL DNS reagent was added and incubated at 50°C for 5 min in water bath [22]. The absorbance was measured at 540 nm. The reaction was terminated at zero time in the control tubes. The standard graph was prepared using 0–500 *μ*g xylose. An autozero was set in UV-VIS spectrophotometer (Hitachi, Japan) using buffer solution. One unit of xylanase activity was defined as the amount of enzyme that liberates 1 micromole of reducing sugars equivalent to xylose per minute under the assay conditions described. Solubilised xylan was prepared by stirring birchwood xylan with 1 M NaOH for six hours at room temperature followed by centrifugation and freeze drying the supernatant after neutralising the alkali with 1 M HCl.

### 2.7. Cellulase Assay

Cellulase activity was measured according to Ghose with necessary modifications [23]. A 900 *μ*L 1% carboxy methyl cellulose solution was added with 100 *μ*L enzyme in a test tube. 1.5 mL DNS reagent was added and incubated at 50°C for 5 min in water bath. The absorbance was measured at 540 nm. The reaction was terminated at zero time in control tubes. A standard graph was prepared using 0–500 *μ*g glucose. An autozero was set in spectrophotometer using buffer solution. One unit of cellulase activity was defined as the amount of enzyme that liberates 1 micromole of glucose equivalents per minute under the assay conditions.

### 2.8. 1,4-*β*-xylosidase Assay

1,4-*β*-xylosidase activity was measured according to Lachke [24]. A 900 *μ*L *p*-nitrophenyl *β*-xyloside (**ρ**-NPX) solution was added with 100 *μ*L of appropriately diluted enzyme solution in a test tube. The mixture was incubated at 50°C for 30 min. Then 1 mL of 2 M sodium carbonate solution was added. The absorbance was measured at 410 nm. The reaction was terminated at zero time in control tubes. One unit of 1,4-*β*-xylosidase activity was defined as the amount of enzyme that catalyzes the formation of 1 micromole of **ρ**-nitrophenol per minute under assay conditions.

### 2.9. Determination of Total Protein Content

Total soluble protein was measured according to Lowry et al. [25]. Protein concentration was determined using bovine serum albumin (BSA) as a standard. The protein content of the chromatographic eluant was measured by monitoring the optical density at 280 nm.

### 2.10. Ammonium Sulphate Precipitation

Protein precipitation by salting out technique using ammonium sulphate (NH_4_(SO_4_)_2_) was carried out with constant gentle stirring [26]. This was left overnight and the precipitate was collected by centrifugation at 10,000 ×g for 10 min. The precipitate obtained was dissolved in phosphate buffer (50 mM, pH 8.0) and dialyzed against the same buffer for 24 h. Dialysis was carried out using cellulose tubing (molecular weight cut-off 13,000 kDa, Himedia LA393-10 MT).

### 2.11. Ion Exchange Chromatography

Dialyzed enzyme (2 mL) was loaded onto a anion exchange DEAE Cellulose (Sigma-Aldrich Co., USA) column. The column was packed with activated DEAE-cellulose equilibrated with 50 mM phosphate buffer (pH 8.0). The height of column was 20 cm with the 2.5 cm diameter. The protein was eluted with the 0.0 to 0.5 M NaCl gradient. The 50 fractions were collected having 5 mL volume of each fraction with the flow rate of 1 mL/min. All the steps were carried out at 4 to 8°C.

### 2.12. Molecular Mass Determination by SDS-PAGE

SDS-PAGE of partially purified xylanase was performed in a 12.5% acrylamide gel Laemmli [27]. Coomassie brilliant blue R-250 was used to stain the gel. The protein molecular weight markers used were of medium range containing 14.4 kDa to 94.0 kDa obtained from Bangalore GeNei, India.

### 2.13. Substrate Specificity

Substrate specificity of the xylanase was found by using 1% xylan, cellobiose, starch, carboxy methyl cellulose (CMC), and *p*-nitrophenyl xylopyranoside and Avicel as substrates.

### 2.14. Kinetic Parameters

Initial reaction rates using birchwood and oat spelt xylan as substrate were determined at substrate concentrations of 0.5–10 mg/mL in 50 mM phosphate buffer (pH 7.0) at 45°C. The kinetic constants, Km and Vmax, were estimated using the linear regression method of Lineweaver and Burk [28].

### 2.15. Identification of Hydrolysis Products

To 50 mL of birchwood xylan suspension (1% of birchwood xylan in 50 mM Phosphate buffer pH 7.0), 40 *μ*g of xylanase enzyme was added and incubated at 45°C. Hydrolysis products were detected by thin layer chromatography (TLC) [29]. TLC (TLC plates, 0.25 mm layers of silica gel F 254, Merck, India) was performed using the mixture of *n*-butanol : ethanol : H_2_O (5 : 3 : 2 by vol) as a solvent system. Compounds were detected by spraying with 50% sulphuric acid in ethanol followed by heating at 150°C for 5 min. D-xylose (X_1_), xylobiose (X_2_), xylotriose (X_3_), and xylotetraose (X_4_) were applied as standard.

### 2.16. Effect of Temperature on Activity and Stability

The optimum temperature for maximum xylanase activity was determined by varying the reaction temperature from 30 to 80°C. To evaluate thermal stability, 0.5 mL of the enzyme solution was incubated at 30–80°C temperatures for up to 4 h. The relative enzyme activity was recorded at 1 h interval during period of 4 h.

### 2.17. Effect of pH on Activity and Stability

The effect of pH on enzyme activity was determined by incubating xylanase at various pH ranging from 6.0 to 11.0. The various buffers used were 50 mM sodium phosphate (pH 6, 7), 50 mM Tris HCl (pH 8, 9), 50 mM carbonate bicarbonate buffer (pH 10), and 50 mM glycine-NaOH buffer (pH 11). To evaluate the stability of the enzyme at each pH, the purified enzyme was incubated into the respective buffer over a pH range of 6.0–11.0 for up to 4 h at optimum temperature. The relative enzyme activity was determined at 1 h interval during the 4 h period of incubation.

## 3. Results and Discussion 

### 3.1. Isolation and Identification of Bacteria

**Figure 1:**
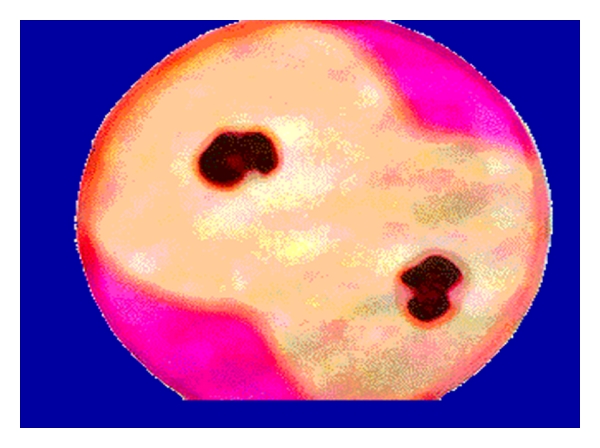
Plate showing zone of clearance around colony by isolate.

About 25 bacterial strains, which formed clear halos around their colonies on xylan agar plates, were picked up for further studies, isolated from soil collected at selected study site. The strain that showed 33 mm zone of clearance around the colony proved its xylanolytic ability (Figure 1). It was identified on the basis of various morphological and biochemical characteristics as shown in Table 1.

**Figure 2:**
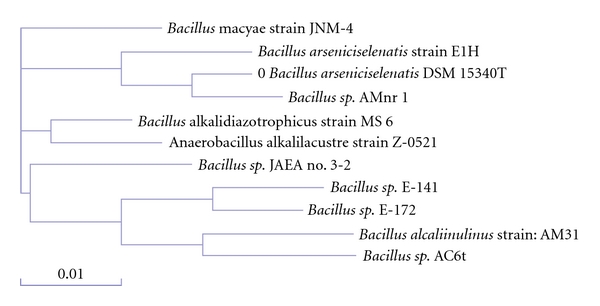
The phylogenetic tree of *Bacillus arseniciselenatis *DSM 15340 (designated as “0”).

The isolate was confirmed as *Bacillus arseniciselenatis *strain DSM-15340 with partial 16S rRNA sequencing having a length of 1499 bp nucleotide. The sequence was deposited in Gene Bank (Accession No. AJ865469). The phylogenetic relation of this isolate is as shown in Figure 2. It is closely associated with *Bacillus sp.* AMnr. It was also isolated from soil sample collected at coastal areas of Mandovi, Goa. Shivaji et al. isolated *Bacillus arseniciselenatis *DSM 15340 and *Bacillus arsenicus* from a bore well located in the chakdah region of West Bengal, India [30]. 

### 3.2. Xylanase Production in SSF

When the strain was grown on wheat bran for 3 days of incubation at pH 8.0 and 45°C, maximum xylanase production was observed, that is, 910.49 U/gram dry substance, which was absolutely free from cellulase. Several workers reported the suitability of wheat bran for xylanase production in SSF [31, 32]. Commercial wheat bran consists of 30% cellulose, 27% hemicellulose, 21% lignin, and 8% ash [33]. Hence there was increase in possibility of cellulase contamination when grown on wheat bran. Haltrich et al. also reported that xylanases were always associated with cellulase [34]. From twenty selected strains, five were able to produce cellulase along with xylanase in SSF. This was due to the presence of cellulose in substrate wheat bran used in SSF. 

### 3.3. Purification of Xylanase

**Figure 3:**
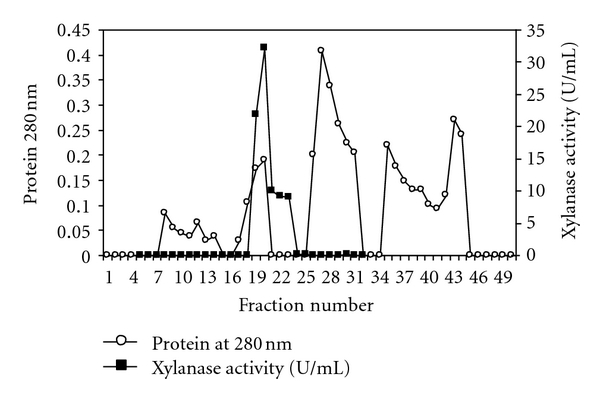
Elution profile of xylanase from DEAE-cellulose column chromatography.

The culture filtrate was precipitated by fractional (35–80%) ammonium sulphate saturation. Proteins precipitated within this range had maximum xylanase activity and was used for purification. Xylanase was further purified by DEAE cellulose ion exchange column. The enzyme was eluted from DEAE cellulose column at a NaCl concentration of 0.25 M (Figure 3). The fractions (no. 19–25) having maximum specific activity were concentrated. Xylanase was purified 3.06-fold with a specific activity of 299.25 U/mg (Table 2). 

The specific activity of xylanase produced by *Bacillus pumilus* was previously reported as 298 U/mg by Panbangred et al. [35].

### 3.4. Molecular Weight Determination

**Figure 4:**
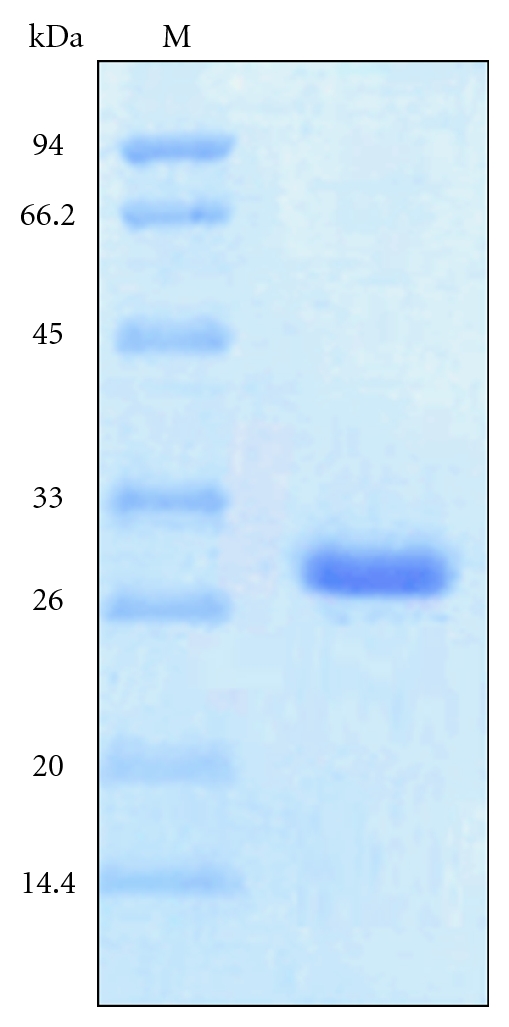
SDS-PAGE analysis of purified xylanases from *Bacillus arseniciselenatis* DSM 15340. Lane M: molecular markers; Lane B: Purified xylanase enzyme.

The purified enzyme showed a single-protein band on SDS-PAGE. The molecular mass of denatured xylanase, estimated from the relative mobility of proteins on SDS-PAGE, was ~29.8 kDa as shown in Figure 4. The present results were supported by previous work. The enzyme from a fungus *Plectosphaerella cucumerina *had a molecular weight of 19 kDa reported by Zhang et al. [36]. Xylanase produced by *Bacillus sp*. strain BP-23 is of 32 kDa [37] whereas the second xylanase obtained from *Bacillus firmus *had a molecular weight of 45 kDa [38]. 

### 3.5. Substrate Specificity

The action of the purified xylanase towards various substrates was studied. The enzyme was active on birchwood xylan, little active on *p*-nitrophenyl xylopyranoside but not on Avicel, CMC, cellobiose, and starch (Table 3). Purified xylanase was not active on Avicel, CMC, cellobiose, and starch even when the enzyme concentration was 5 times greater than used in normal assay and incubation period of 20 minutes rather than 5 minutes. Similarly, xylanase with absolute substrate specificity was purified from *Trichoderma viride* by Ujiie et al. [39]. Kanda et al. purified two different xylanases, named xyl I and III that showed no activity towards glycans, other than xylan, such as starch, pachyman, and Avicel (microcrystalline cellulose), except for the almost one twentieth activity of xyl III toward carboxymethyl cellulose (CMC) [40].

### 3.6. Kinetic Parameters

**Figure 5:**
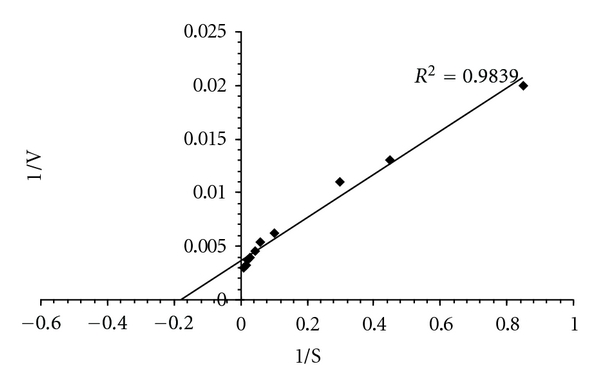
Double reciprocal plot for determining the *V*_max⁡_ and Km values of xylanase *Bacillus arseniciselenatis* DSM 15340 when acted on Birchwood xylan.

The kinetic parameters Km and *V*_max⁡_ of the enzyme were determined from Lineweaver-Burk double-reciprocal plots of xylanase activity at 45°C using various concentrations of birchwood xylan as substrate (Figure 5). The Km and *V*_max⁡_ values of xylanase were 5.26 mg/mL and 277.7 *μ*mol/min/mg, respectively. Wang et al. reported that Km and *V*_max⁡_ values of xylanase isolated from *Bacillus sp*. NTU-06 were 3.45 mg/mL and 387.3 *μ*mol/min/mg, respectively [41]. Bansod et al. also reported that Km values of xylanases lie in the range from 0.5 to 19.6 mg/mL [42]. Xylanases isolated from *Aeromonas cavie *171 ME-1 and *Bacillus sp. *strain 41m-1 showed similar values of *V*_max⁡_ 260 to 350 *μ*mol/min/mg protein [43, 44].

### 3.7. Analysis of Hydrolytic Products

**Figure 6:**
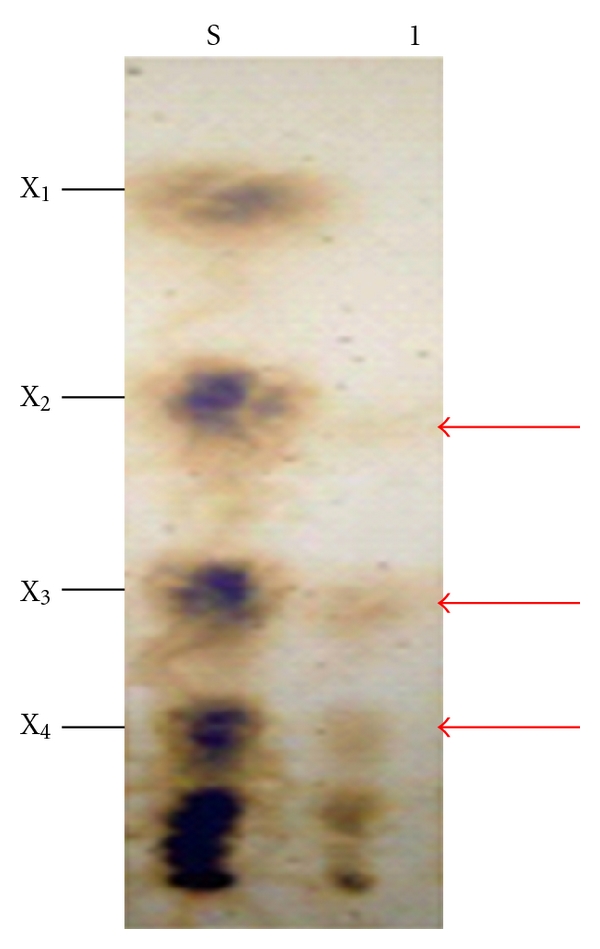
TLC analysis for hydrolysis products released from birchwood xylan by xylanase from *Bacillus arseniciselenatis* DSM 15340. S: substrate; 1: sample; X_1_: D-xylose; X_2_: xylobiose; X_3_: xylotriose; X_4_: xylotetraose.

After 1 h of incubation of birchwood xylan with xylanase *Bacillus arseniciselenatis* DSM 15340, xylotriose and xylotetraose were the main products in the hydrolytic mixture along with little amount xylobiose. (Figure 6). The present results indicated that xylanase cleaved the substrate to liberate mainly xylooligosaccharides, but not able to act on resulting oligosaccharides to form xylose, suggesting that it is a endoxylanase.

Analysis of hydrolytic products of xylan by the xylanase of *Thermoascus aurantiacus* showed that xylan was degraded to various xylo-oligosaccharides without a significant accumulation of xylose [45]. Xylobiose and xylotriose were the main hydrolysis products when xylanase of *Bacillus stearothermophilus* reacted with oat spelt xylan and resulted oligosaccharides were then cleaved to form xylose by the *β*-xylosidase action [46]. The end products were xylobiose, xylotriose, xylotetraose, and higher oligosaccharides when xylan was hydrolyzed with endoxylanase of alkalophilic *Bacillus sp.* No. C-125. No xylose was found in the hydrolysis products when analysed by HPLC [47]. 

### 3.8. Effect of Temperature on Activity and Stability

**Figure 7:**
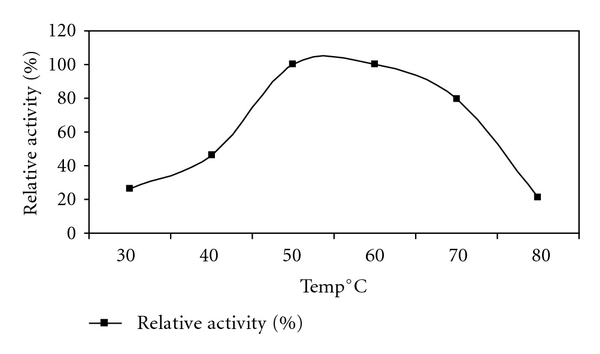
Effect of temperature on activity of xylanase from *Bacillus arseniciselenatis* DSM 15340.

**Figure 8:**
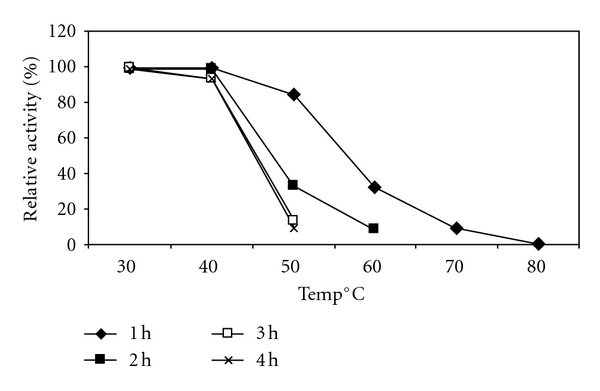
Effect of temperature on stability of xylanase from *Bacillus arseniciselenatis* DSM 15340.

For xylanase from *Bacillus arseniciselenatis* DSM 15340, activity was found to be gradually increased with increasing temperature and found significantly declined at 80°C (Figure 7). 50°C was found to be the most favourable for enzyme activity. Stability of the enzyme was the most important factor in studying characteristics. In case of xylanase purified from *Bacillus arseniciselenatis* DSM 15340, it was more stable at temperatures 30°C and 40°C for 4 h of incubation and retained almost 93% activity. At higher temperature values xylanase stability was gradually declined (Figure 8). Bernier et al. reported that multiple forms of xylanases were purified from *Aeromonas sp.* by Ohkoshi et al. and the properties of the three xylanases were well characterized [2, 48]. It was found that these xylanases were most active at 50°C to 60°C. Kang et al. purified two xylanases which gave the highest activity at 50°C. They showed relatively high stabilities at 50°C temperature [49].

### 3.9. Effect of pH on Activity and Stability

**Figure 9:**
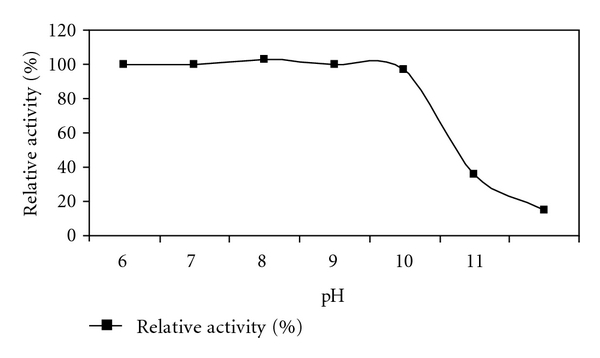
Effect of pH on activity of xylanase from *Bacillus arseniciselenatis* DSM 15340.

**Figure 10:**
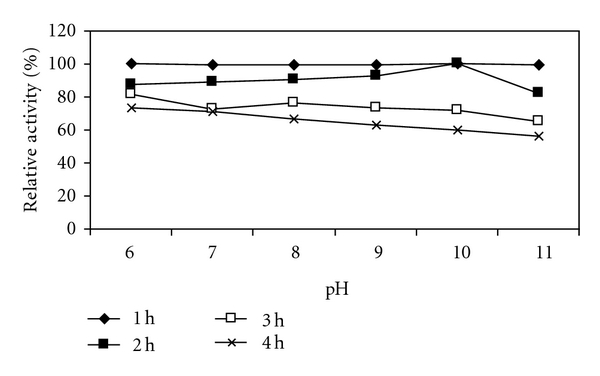
Effect of pH on stability of xylanase from *Bacillus arseniciselenatis* DSM 15340.

pH was the most important factor to characterize the enzyme. Xylanase from *Bacillus arseniciselenatis *DSM 15340 showed 100% activity at pH 8.0 (Figure 9). At higher pH values also, activity was 95%. With respect to stability, at all tested pH values xylanase activity was 100% activity for 1 h. pH 10.0 was the most favourable for stability and retained 60% activity for 4 h of incubation (Figure 10).

Similarly, Honda et al. purified two xylanases namely xylanases N and xylanase A from *Bacillus sp.* No. C-125 [47]. Among these xylanases, N shows maximum activity at pH ranging from 6.0 to 7.0, while xylanase A was active at pH ranging from 6.0 to 10.0 and showed some activity at pH 12.0 also. In the present study, 100% activity was retained by* Bacillus arseniciselenatis *DSM 15340 xylanase for 2 h of incubation at pH 10.0. Stability at the extreme pH values may be due to charged amino acid residues. The enzymes stable in alkaline conditions were characterized by a decreased number of acidic residues and an increased number of arginines [50].

## 4. Conclusions

*Bacillus arseniciselenatis* DSM 15340 produced a thermoalkalophilic cellulose-free xylanase in higher amount when grown on solid state conditions using cheaply available agroresidual substrate wheat bran. Hence it can be used for large-scale production of xylanase using such agroresidual substrates. The purified xylanase also was capable of producing high-quality xylo-oligosaccharides, indicating its application potential not only in pulp biobleaching processes but also in the nutraceutical industry.

## Figures and Tables

**Table 1:** Morphological, physiological, and biochemical characteristics of the isolate.

Tests	Results	Tests	Results
Colony morphology		Growth on NaCl (%)	
Shape	Circular	2.0	−
Margin	Regular	4.0	−
Elevation	Raised	6.0	+
Consistency	Moist	8.0	+
Color	Pale yellow	10.0	−
Opacity	Opaque	Anaerobic Growth	Facultative
Gram nature	Gram Positive	Utilization of carbohydrates	
Shape of the cell	Long rods	Xylose	+
Motility	Sluggish Motile	Lactose	+
Endospore position	Central	Mannitol	−
Growth at temperatures		Arabinose	−
10°C	−	Sucrose	−
25°C	−	Glucose	−
30°C	+	Fructose	+
37°C	+	Melibiose	−
45°C	+++	Starch hydrolysis	+
60°C	+	Gelatin hydrolysis	+
70°C	–	Urea hydrolysis	−
Growth at pH		Esculin hydrolysis	−
5.0	−	Casein hydrolysis	−
6.0	−	Tween 20 hydrolysis	+
7.0	+	Catalase test	+
8.0	++	Oxidase test	+
10.0	−	Nitrate reduction	+
		H_2_S production	−

+: Positive; −: Negative.

**Table 2:** Purification steps of xylanase enzyme isolated from *Bacillus arseniciselenatis* DSM 15340 when grown on wheat bran.

Purification steps	Xylanase activity (U)	Total protein content (mg)	Specific activity (U/mg)	Purification fold
Crude filtrate	231659	2376	97.49	1.0
(NH_4_)_2_SO_4_ precipitation	196220	1460	134.39	1.37
DEAE sepharose FF	96360	322	299.25	3.06

**Table 3:** Substrate specificity of purified xylanase.

Substrates	Xylanase activity (U/mg protein)
Birchwood xylan	291.9 + 0.35
Cellobiose	0.0 + 0.0
Starch	0.0 + 0.0
Carboxy methyl cellulose (CMC)	0.0 + 0.0
*p*-Nitrophenyl xylopyranoside	0.22 + 0.001
Avicel	0.0 + 0.0

Each value represents the mean + standard error values.
